# Modification and Targeted Design of N-Terminal Truncates Derived from Brevinin with Improved Therapeutic Efficacy

**DOI:** 10.3390/biology9080209

**Published:** 2020-08-06

**Authors:** Haoyang He, Yuqing Chen, Zhuming Ye, Xiaoling Chen, Chengbang Ma, Mei Zhou, Xinping Xi, James F. Burrows, Tianbao Chen, Lei Wang

**Affiliations:** Natural Drug Discovery Group, School of Pharmacy, Queen’s University Belfast, Belfast BT9 7BL, Northern Ireland, UK; hhe06@qub.ac.uk (H.H.); ychen52@qub.ac.uk (Y.C.); zye04@qub.ac.uk (Z.Y.); x.chen@qub.ac.uk (X.C.); m.zhou@qub.ac.uk (M.Z.); j.burrows@qub.ac.uk (J.F.B.); t.chen@qub.ac.uk (T.C.); l.wang@qub.ac.uk (L.W.)

**Keywords:** amphibian skin secretion, antimicrobial peptide, structure-activity relationship, hydrophobicity

## Abstract

Antimicrobial peptides (AMPs) are a class of molecules that play an essential role in innate immune regulation. The Brevinin-1 family are AMPs that show strong pharmacological and antimicrobial potential. A novel peptide, B1A, was designed based on the primary structure of brevinin-1PLb and brevinin-1PLc. Subsequently, a synthesised replicate was subjected to a series of bioassays and was found to display antimicrobial activity. However, it also displayed high levels of haemolysis in a horse red blood cell haemolytic assay, suggesting potential toxicity. Therefore, we rationally designed a number of B1A analogues with aim of retaining antimicrobial activity, lowering toxicity, and to explore the structure–activity relationship of its N-terminus. B1A and its analogues still retained the “Rana Box” and the FLP-motif, which is a feature of this subfamily. However, the introduction of Lys and Trp residues into the peptide sequences revealed that antimicrobial activity of these analogues remained unchanged once the hydrophobicity and the charge reached the threshold. Hence, the idea that the hydrophobicity saturation in different situations is related to antimicrobial activity can be understood via the structure–activity relationship. Meanwhile, it could also be the starting point for the generation of peptides with specific antimicrobial activity.

## 1. Introduction

Development of antimicrobial resistance leads to the failure of clinical treatment using traditional antibiotics [1]. For decades, antimicrobial peptides (AMPs), discovered among all classes of living organisms from bacteria, plants, invertebrate, and vertebrate species [2], have attracted attention as potential novel antimicrobial alternatives [3]. Generally, AMPs consisting of 10 to 50 amino acids, which are cationic and amphipathic, have shown the ability to kill Gram-negative and Gram-positive bacteria, viruses, fungi, and cancer cells [4].

Amphibian skin secretions are a remarkable source of potentially novel therapeutic agents [5], and amphibian AMPs have demonstrated outstanding efficacy and kill microorganisms via a number of different mechanisms, offering the prospect of avoiding antibiotic-resistance [6]. The Brevinin-1 peptide family is one of the most ubiquitous AMP families [7], and since the first Brevinin-1 peptide with broad-spectrum antimicrobial activity was isolated from *Rana brevipoda porsa*, 985 Brevinin-1 peptides have been deposited into the database [8]. They share a highly conserved C-terminal disulphide bridged cyclic heptapeptide loop, namely the “Rana Box” [9]. Studies revealed that eliminating the disulphide bridge does not alter the biofunction remarkably [10], but changes to the amino acid constitution of the loop domain have a significant impact on the biological activity of brevinin-1 peptides [10,11,12]. However, a study on Brevinin-1E has shown the disulphide bridge is necessary for structural stability, but moving the “Rana box” loop from the C-terminus to the middle of the peptide did not significantly affect the antimicrobial activity [13].

In addition, brevinin-1 peptides have a hydrophobic N-terminal domain that is highly conserved and includes an FLP-tripeptide at the N-terminus and a Pro hinge at position 14 [10]. Reducing the hydrophobic domain significantly decreases antimicrobial and haemolytic activity [10], while changing the Pro to Ala at position 14 has been found to increase the associated cytotoxicity due to the enlargement of the helical domain [14].

Brevinin-1PLb and brevinin-1PLc are two previously identified brevinin-1 peptides discovered from the skin secretions of *Lithobates palustris* (*Rana palustris*), and both peptides exhibit broad-spectrum and similar antimicrobial activity [15]. Their biosynthetic precursor encoding cDNAs were subsequently verified by our group [16]. Similarly, they possess the hydrophobic N-terminal domain with the typical FLP and proline hinge motifs as well as the C-terminal heptapeptide loop. Notably, they have two FLP motifs that may contribute to the activity in the same way [14]. Here, we employed these brevinin-1 peptides as templates to design N-terminal truncated analogues with high efficacy and elucidate the structure–activity relationship of the N-terminal domain of brevinin-1 peptides.

## 2. Materials and Methods

### 2.1. Fmoc Solid-Phase Peptide Synthesis

After the authentic and unequivocal sequence was obtained, peptides (B1A and its analogues) were synthesised via the Solid Phase Peptide Synthesis (SPPS) as described previously [17]. Briefly, the protected group of each amino acid was removed by piperidine/N, N-dimethylformamide (DMF) (20/80, *V*/*V*). The formation of peptide chain was accomplished by 2-(1H-benzotriazole)-1, 1, 3, 3-tetramethyluronium (HBTU) in 1 M 4-methylmorpholine (NMM)/DMF solution. After that, the resin of peptide was removed by the cleavage mixture with trifluoroacetic acid (TFA), water, thioanisole (TIS), and 1, 2-ethanedithiol (EDT) (94/2/2/2; *V*/*V*/*V*). Hydrogen peroxide (H_2_O_2_) was executed to oxidise linear peptides as described previously [18]. Disulphide bridges were formed in the presence of 0.5% (*w*/*v*) H_2_O_2_ for 0.5 h at room temperature before lyophilisation. Each peptide was verified by LC-MS analysis and purified by RP-HPLC (Figure S1).

### 2.2. Secondary Structure Prediction and Verification

The predicted physicochemical properties (sequence, amino acid number, hydrophobicity, hydrophobic moment, and net charge) and the helical wheel projections of all peptides were obtained via Heliquest [19]. The determination of the secondary structure of peptides was conducted using a JASCO J-815 CD Spectropolarimeter (JASCO Inc., Tokyo, Japan). The circular dichroism (CD) spectrum results were analysed by the BeStSel online server for the determination of α-helix and turn content [20]. The 3D models were predicted via I-TASSER online server (University of Michigan, Ann Arbor, MI, USA) [21].

### 2.3. Antimicrobial Assay

The antimicrobial assay was carried out to detect the minimum inhibitory concentration (MIC) and the minimum bactericidal concentration (MBC) of each peptide on the microbes as described previously [22]. Seven different microorganisms were used in the assay: Gram-positive bacteria *Staphylococcus aur*eus (NCTC10788), *Enterococcus faecalis* (NCTC12697), and methicillin-resistant *S. aureus* (MRSA) (NCTC12493); Gram-negative bacteria *Escherichia coli* (NCTC10418), *Pseudomonas aeruginosa* (ATCC27853), and *Klebsiella pneumoniae* (ATCC 43816); and fungi *Candida albicans* (NCYC1467). Bacterial strains were inoculated and grown to mid-log phase in fresh Mueller Hinton broth (MHB) at 37 °C for overnight, then bacteria inoculum suspensions were subcultured until reaching the logarithmic growth phase by measuring OD of the cultures at wavelength 550 nm. Then, 99 µL bacteria and 1 µL peptide solution were mixed in the 96-wells microplate. For positive and negative controls, the bacteria were incubated with antibiotics (Norfloxacin for Gram-positive bacteria and Gram-negative bacteria; Amphotericin B for fungi) solution and MHB medium, respectively. Experiments were repeated five times in five replicates.

### 2.4. Haemolytic Assay

The haemolytic assay was carried out to detect the cytotoxicity of each peptide to erythrocytes obtained from fresh defibrinated horse blood as described before [23]. An appropriate volume of horse blood solution was collected via washing and transferring until the supernatant was clear. Then, 2% (*v*/*v*) suspension of red blood cells were incubated with peptide at 37 °C for 2 h. For positive and negative controls, the red blood cells were incubated with 1% (*v*/*v*) Triton-X 100 and Phosphate-buffered saline (PBS) solution, respectively. The 10% of maximal haemolysis concentration (HC_10_, μM) was utilised to assess the haemolytic potential. Experiments were repeated three times in five replicates.

### 2.5. SYTOX Green Uptake Assay

The SYTOX Green uptake assay was accomplished to detect the cell membrane permeability abilities of each peptide as described previously [22]. The bacterial strains were inoculated and grown to mid-log phase in fresh tryptic soy broth (TSB) medium (Sigma-Aldrich, St. Louis, MO, USA) at 37 °C overnight, and then bacteria inoculum suspensions were subcultured for 2.5 h. Bacterial cells were collected and centrifuged at 1000× *g* for 10 min at 4 °C followed by washing with 5% TSB in 0.85% NaCl solution twice. The precipitates of bacterial cells were resuspended with 5% TSB in 0.85% NaCl solution to obtain a cell concentration of 1 × 10^8^ CFU/mL by measuring OD_590_ of 0.70. The strains were incubated with peptide in several concentrations based on the MIC results. The strains were stained with SYTOX GREEN nucleic acid stain (Thermo Fisher Scientific, Waltham, MA, USA) dissolved in 5% TSB in 0.85% NaCl to a final concentration of 5 µM. The fluorescent intensity was detected with a Synergy HT plate reader (Biotech, Minneapolis, MN, USA). The fluorescence was recorded as a function of time for 2 h. For positive and negative controls, the bacteria were incubated with 32 µM Melittin solution and 0.85% NaCl solution containing 5% TSB, respectively. Experiments were repeated three times in five replicates.

### 2.6. Outer Membrane Permeability Assay

The outer membrane permeability activity against Gram-negative bacteria *E. coli* was determined by the uptake of N-phenyl-1-naphthylamine (NPN) [24,25]. Briefly, *E. coli* grew to the logarithmic phase (OD_550_ = 0.42) at 37 °C in lysogeny broth (LB) medium. Then, bacterial cells were harvested by centrifugation at 1000× *g* for 10 min at 4 °C. The cells were washed twice with 5 mM of 4-(2-hydroxyethyl)-1-piperazineethanesulfonic acid (HEPES) buffer containing 5 mM glucose (pH = 7.2) and resuspended to an OD_550_ of 0.2. 100 µL *E. coli* and 50 µL peptide solution were mixed with 50 µL NPN (final concentration, 10 µM). The positive control group was carried out with ethylenediaminetetraacetic acid (EDTA), and the negative control group was carried out with HEPES buffer. Black wall 96-well plates were monitored using a microplate reader (Biolise BioTek EL808, Winooski, VT, USA) at excitation and an emission wavelength of 350 nm and 420 nm, respectively. Experiments were repeated three times in five replicates.

### 2.7. Inner Membrane Permeability Assay

The inner membrane permeability activity against *E. coli* bacteria was determined by the uptake of Ortho-Nitrophenyl-β-D-glucopyranoside (ONPG) as reported before [26,27]. Briefly, *E. coli* were grown to the logarithmic phase (OD_550_ = 0.42) at 37 °C in LB medium containing 2% lactose. Then, bacterial cells were harvested by centrifugation at 1000× *g* for 10 min at 4 °C. The cells were washed twice with PBS and suspended with 10 mM PBS buffer (pH 7.4) that contained 1.5 mM ONPG to an OD_600_ of 0.05. Then, 99 µL *E. coli* and 1 µL peptide solution were incubated with 99 µL cell culture in each well of the 96-well plate. The positive control group was executed with a 32 µM Melittin solution, and the negative control group was executed with PBS buffer. The plate was detected using a microplate reader (Biolise BioTek EL808, Winooski, VT, USA) at an excitation and an emission wavelength of 350 nm and 420 nm, respectively. Experiments were repeated three times in five replicates.

### 2.8. Statistical Analysis

Statistical analysis was executed by the GraphPad Prism 6 (GraphPad Software, San Diego, CA, USA). Data were shown with the mean values ± SD of five replicates.

## 3. Results

### 3.1. Rational Design of Novel Peptide

**Figure 1:**
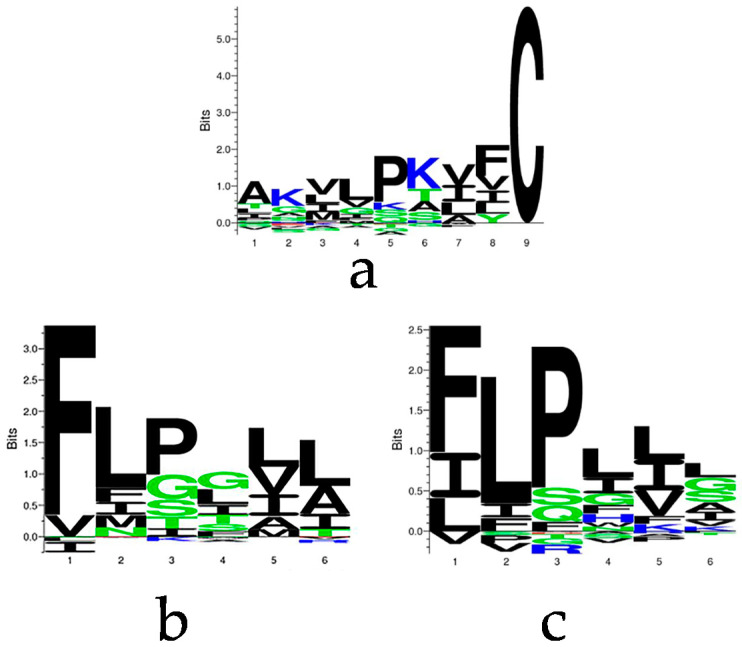
Motif analysis of the selected sequences from Antimicrobial Peptide Database (APD3). The sequence cluster of (**a**) 9 amino acid residues before the rana box domain of 121 brevinin-1 peptides, (**b**) the N-terminal 6-mer peptide of selected brevinin-1 peptides, and (**c**) the N-terminal 6-mer peptide of 102 temporin peptides. The hydrophobic and positively charged amino acids are coloured by black and blue, respectively.

The characteristics of brevinin-1 peptides have been generally summarised into several domains, including a hydrophobic N-terminus and C-terminal rana box. To further elucidate the amino acid constitution, motif analysis using the sequences deposited in the Antimicrobial Peptide Database (APD3) was performed by Seq2Logo algorithm. Analysis of the nine amino acid residue domain in front of the rana box loop (the “C” at position 9 in Figure 1a indicates the first Cys of rana box domain) of 116 brevinin-1 sequences demonstrated that the amino acid constitution varied greatly, although most of them were hydrophobic, whilst the N-terminal tripeptide, FLP, of brevinin is highly conserved with the temporin peptide family, an AMP family from the skin secretions of Ranidae frogs (Figure 1b,c).

Brevinin-1PLb (B1PLB) and brevinin-1PLc (B1PLC) exhibited high degrees of sequence identity, and both possessed potent antimicrobial activity against *S. aureus*, *E. coli*, and *C. albicans* [15]. Although B1PLC had better activity against bacteria, its potency was slightly lower than that of B1PLB against fungi. As we know, the more positive charge of B1PLC (Table 1) could contribute to an enhanced electrostatic interaction with the negatively charged bacterial cell envelope. Therefore, to try to combine the properties of the two peptides, we designed B1A by substitution of the Asn residue at position 11 in B1PLB with Lys. 

In addition, B1PLB and B1PLC possess an additional FLP domain in the middle of their sequence, which is not a structural feature but is often observed in other brevinin-1 peptides. Therefore, to explore the potential function of the FLP domains, we designed two truncated analogues, B1A1 and B1A2, which had one of the two FLP segments removed and thus had either FLPLIAGLAAK or FLPKIF attached to the Rana box (Table 1). Both truncated peptides showed a marked decrease in antimicrobial activity as well as haemolytic activity. However, B1A2 retained weak antimicrobial activity due to the presence of the helical domain after the proline hinge. In comparison with the parent peptide, the hydrophobicity and the charge of B1A2 were drastically reduced. Thus, more analogues with enhanced charge and hydrophobicity were designed by introducing Lys residues or substituting Lys for Trp.

The analogue KB2 was created by adding a Lys residue onto the N-terminus of B1A2, and KKB2 was created by adding an additional Lys to further alter the charge. The next rationally designed analogues altered the hydrophobicity by inserting a Trp at position 5 of KB2 to create KWB2. Two further analogues, KW^3,5^B2 and KW^5,7^B2, were designed by replacing Ile^3^ and Leu^7^ with Trp in KWB2, respectively. Meanwhile, the role of hydrophobicity was examined in the same way on KKB2 by adding a Trp at position 6 (KKWB2). All these analogues share the family features (Table 1), the FLP motif and C-terminal “Rana Box”.

### 3.2. Secondary Structure Analysis

**Figure 2:**
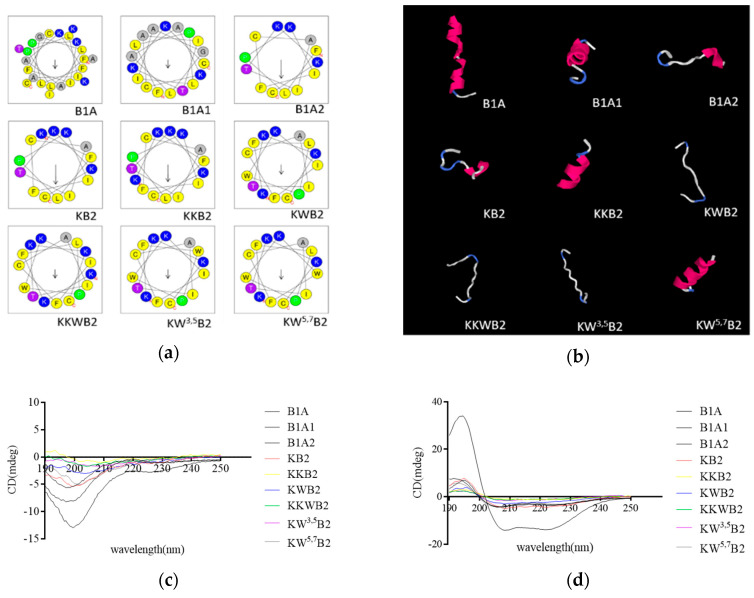
(**a**) Helical wheel representation of all peptides. (**b**) Predicted 3D models of all peptides circular dichroism (CD) spectra of B1A and its analogues. The peptides were tested in aqueous solution (**c**) and 1% SDS micelles solution (**d**), respectively.

To alter the hydrophobicity of these peptides, Trp was inserted into the nonpolar surface to enhance the amphiphilicity (Figure 2a). B1A showed a very low propensity to form a proper alpha-helical conformation in the aqueous solution. However, it showed a very high-proportion of α-helix conformation in 1% SDS micelle solution, which is similar to the bacteria membrane environment, with the helix content of B1A estimated to represent about 66.7% of the secondary structure (Table 2). Compared with the parent peptide, the eight analogues (Figure 2c,d) showed a low proportion of α-helical conformation in a 1% SDS micelle solution and the same random coil in aqueous solution. The secondary structure prediction via I-TASSER verified the analysis of the secondary structure to some extent (Figure 2b).

### 3.3. Cytotoxicity

**Figure 3:**
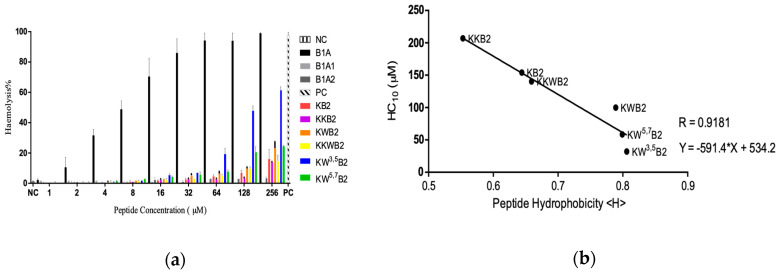
(**a**) The haemolytic activity of novel peptides (B1A) and its analogues on horse erythrocytes. Phosphate buffered saline (PBS) and 1% TritonX-100 were used as the negative control (NC) and positive control (PC), respectively; (**b**) relationship of peptide’s (KKB2, KB2, KKWB2, KWB2, KW^5,7^B2, and KW^3,5^B2) hydrophobicity and 10% haemolytic complement (HC_10_) on horse erythrocytes. Data represent means ± SD of five replicates.

The haemolytic activity of B1A was quite high, with the maximal concentration causing 10% red cell lysis (HC_10_) being 1.576 µM (Figure 3a). The HC_10_ was in the same order of magnitude as the MIC and the MBC (Table 3). In contrast, the HC_10_ of KKW^3,5^B2 was much higher at 32.07 µM, and the HC_10_s of B1A1 and B1A2 could not be calculated, as it was over the highest concentration of peptide used in these assays (Figure 3a). Although all the analogues’ haemolytic abilities significantly reduced, the differences among them were obvious. The relative hydrophobicities of these peptides was in the order KW^3,5^B2 > KW^5,7^B2 > KWB2 > KKWB2 > KB2 > KKB2, and the relationship between hydrophobicity and the haemolytic assay (Figure 3b) indicated that hydrophobicity ameliorated the haemolysis. Furthermore, the analysis in Figure 3b exhibited that there was a linear relationship between them.

### 3.4. Antimicrobial Activities

The novel peptide B1A displayed potent and broad-spectrum antimicrobial activity (Table 3). However, the various analogues showed much more variability in their potency and in some cases were ineffective.

**Figure 4:**
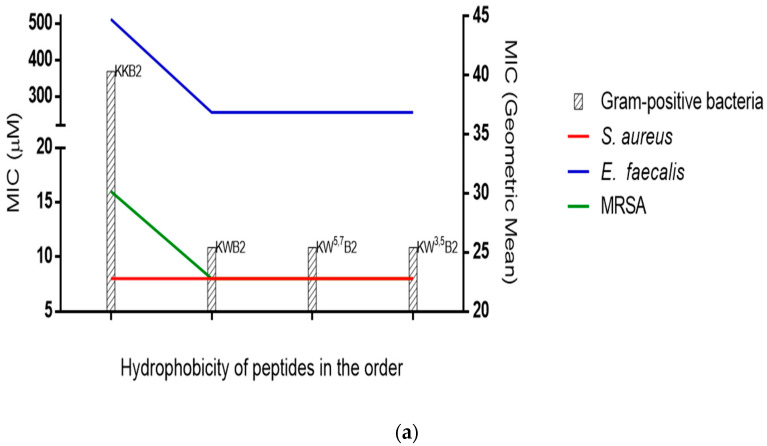

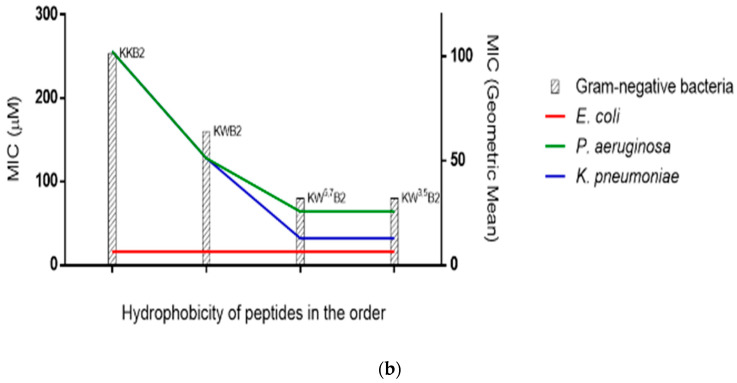
Relationship of peptides’ (KKB2, KWB2, KW^5,7^B2, and KW^3,5^B2) hydrophobicity in the order and MICs. (**a**) The geometric mean of the MICs for three gram-positive bacteria and their respective MICs. (**b**) The geometric mean of the MICs for three gram-negative bacteria and their respective MICs.

Both B1A1 and B1A2 showed a significant decrease in antimicrobial activity compared to the parent B1A peptide and at the concentrations used in these experiments were completely inactive against a number of microorganisms, although B1A2 retained some antimicrobial activity, possibly due to its higher hydrophobicity. KB2 displayed significantly enhanced antimicrobial activity when compared to its parent B1A2 peptide against some bacteria and fungi (*S. aureus*, *E. coli* and *C. albicans*), which demonstrated the potential contribution of charge to antimicrobial activity. Meanwhile, in comparison to KB2, KKB2, which had the additional Lys at the N-terminus, induced a two-fold improvement of antimicrobial activity against *E. coli* and a four-fold improvement against *C. albicans* but showed the same activity against the *S. aureus*. The MICs of KWB2 against *S. aureus* and *E. coli* were the same as those of KB2, but the MIC for *C. albicans* was eight-fold decreased, and there were also improvements against the other Gram-negative bacteria. The further two modifications KW^3,5^B2 and KW^5,7^B2 maintained the same results as KKB2 and KWB2 against *S. aureus* and *E. coli*, while only KW^3,5^B2 had a two-fold improvement compared to KWB2 against *C. albicans*. On the other hand, KKWB2 showed the most potent and broad-spectrum antimicrobial activities among the B1A analogues. The relationship between hydrophobicity and the MIC of four analogues with the same length in Figure 4 indicated a threshold of antimicrobial activity. Among those analogues, KWB2 (Figure 4a) corresponded most closely to the hydrophobic threshold against Gram-positive bacteria, and KW^5,7^B2 (Figure 4b) was closest against Gram-negative bacteria.

In summary, the antimicrobial activities of all the analogues were weaker than that of original peptide B1A. The therapeutic index (TI) was the ratio of HC_10_ and MIC (geometric mean), and KKWB2 had the highest therapeutic index (TI) among all the peptides examined.

### 3.5. The Membrane of Gram-Positive Bacteria

**Figure 5:**
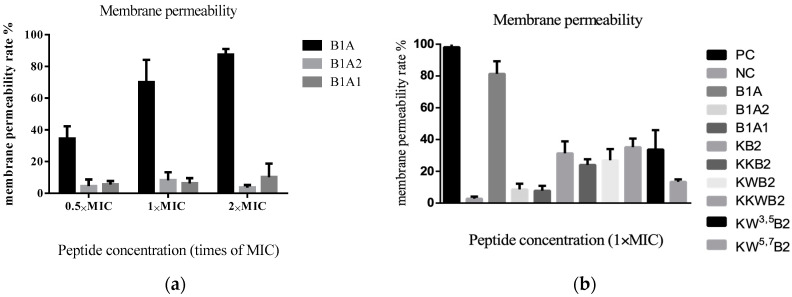
(**a**) Cell membrane effects of B1A, B1A1, and B1A2 on *S. aureus*. (**b**) Cell membrane effects of B1A and its analogues on *S. aureus* with the same concentration. The 0.85% NaCl solution containing 5% tryptic soy broth (TSB) and 32 µM Melittin were used as negative control (NC) and positive control (PC), respectively. Data represent means ± SD of five replicates.

A membrane permeabilisation assay showed that B1A (Figure 5a) thoroughly disrupted the cell membrane of Gram-positive bacteria at 2×MIC. However, the B1A1 and the B1A2 analogues (Figure 5a,b) did not damage the cell membrane up to their respective bactericidal concentrations. None of the other analogues (KB2, KKB2, KWB2, KKWB2, KW^3,5^B2, and KW^5,7^B2) disrupted the membrane to the same extent as B1A but showed similar membrane permeabilisation abilities at 1×MIC (8 µM) against the membrane of *S. aureus.*

### 3.6. The Membrane of Gram-Negative Bacteria

**Figure 6:**
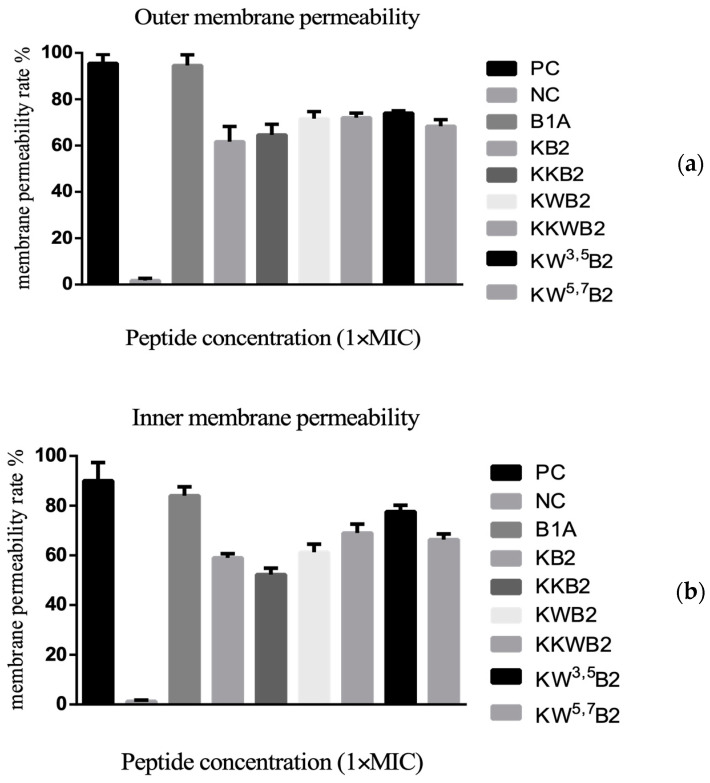
(**a**) Outer membrane permeabilisation assays of E. coli by B1A and six analogues of B1A2 as indicated by the enhanced uptake of NPN. HEPES and EDTA were used as negative control (NC) and positive control (PC), respectively. (**b**) Inter membrane permeabilisation assays of E. coli by B1A and six analogues of B1A2. PBS and 32 µM Melittin were used as negative control (NC) and positive control (PC), respectively. Data represent means ± SD of five replicates.

KB2 and its analogues (Figure 6) induced a similar level of *E. coli* inner and outer membrane permeability at their MICs. These results suggested that, even though the analogues had different antimicrobial abilities, at their MICs, they were all causing membrane permeabilisation.

## 4. Discussion

Novel antimicrobial peptides could be considered as therapeutic agents to solve the complications caused by ongoing diseases and emerging disorders [28]. The Brevinin family is a group of peptides that have been widely studied [7] and found to exhibit antimicrobial activity, especially the brevinin-1 subfamily.

The two truncated analogues, B1A1 and B1A2, showed reduced antimicrobial activity, which might be related to a low degree of helicity and the attenuation of crucial antimicrobial elements such as chain length, charge number, and hydrophobicity [29]. The study of action mechanism assumed that the N-terminal helix could bend and insert into a bilayer to form transmembrane pore, while the C-terminal helix could attach on the lipid surface [30]. A secondary structural study of gaegurin-6, which has a similar sequence to B1A, indicated the Pro produced a stable kink in the molecule [31]. Thus, this feature was retained via the design. 

In regard to antimicrobial activity, there were two parameters (charge and hydrophobicity) considered in the structure–activity relationship studies. Among the extensive reports, amino acid Arg, Lys, and Trp contribute to the antimicrobial activities of AMPs via different steps of the antimicrobial action [32]. An increase of Lys and Arg can enhance the electrostatic interactions with the bacterial membranes [33], whilst introducing Trp as a hydrophobic amino acid can improve the binding ability to the lipids, which may affect the interfacial region of the lipid bilayers [34]. Therefore, this suggests the substitution of Lys and Trp, which increased both the hydrophobicity and the cationic charge of the analogues, would enhance the antimicrobial activity. 

Previous studies have shown that the position of Trp can affect the ability of the peptides to bind the cell membrane [35,36]. Trp plays a unique anchoring role due to its rich content in membrane proteins near the lipid–water interface [29,37,38]. It showed that Trp near the N-terminus enhanced cell binding, while Trp near the C-terminus inhibited this action [39]. Meanwhile, the previous study indicated that Trp close to the N-terminus led to significant haemolytic activity [40]. It explained the remarkable haemolytic ability of KW^3,5^B2 on the other side. The role of Trp was also verified via the enhancement of antimicrobial activity from KB2 to KWB2 and KKB2 to KKWB2 [39]. Furthermore, the helicity of KW^5,7^B2 was slightly higher than that of KW^3,5^B2, which indicated that Trp closer to the N-terminal affected the formation of the helix as reported before [40].

Research on the antimicrobial mechanism of this family indicates Brevinin-1 peptides target the cell membrane [41]. Previous studies have reported a correlation between the haemolytic activity and the hydrophobicity of peptides based on models such as the “barrel-stave” model in eukaryotic cells [42] and the “carpet model” in prokaryotic cells [43]. Peptides can form a pore and penetrate the hydrophobic core of the erythrocyte membrane, causing haemolysis [43]. The relative hydrophobicity of the peptides examined was in the order KW^3,5^B2 > KW^5,7^B2 > KWB2 > KKWB2 > KB2 > KKB2. The haemolytic activities of these analogues were consistent with that positive correlation that the higher the peptide hydrophobicity is, the stronger the haemolytic activity is [44,45,46]. This explained why KW^3,5^B2 exhibited the most vigorous haemolytic activity among the analogues. 

We found that the charge contributed to the antibacterial ability via the antimicrobial activities of two groups: B1A2, KB2, and KKB2; KWB2 and KKWB2. The peptide in each group was designed via the addition of Lys at its N-terminus. However, in the studies looking at hydrophobicity, the results were unexpected. Eliminating interference from other variables, four analogues (KKB2, KWB2, KW^5,7^B2, and KW^3,5^B2) with the same amino acid numbers were analysed. For Gram-positive bacteria (*S. aureus*, *E. faecalis*, and MRSA) without outer-membrane structures, the hydrophobicity of KWB2 was sufficient for maximum antimicrobial activity. There was no further increase in activity with increasing hydrophobicity. Similarly, for Gram-negative bacteria (*E. coli*, *K. pneumoniae*, and *P. aeruginosa*), the hydrophobicity threshold was reached with the analogue KW^5,7^B2. Compared to Gram-positive bacteria, Gram-negative bacteria have a more complex membrane structure, which has an extra outer membrane and an abundance of highly negatively charged lipopolysaccharide [47,48]. The peptides permeabilised the outer membrane and the inner membrane, and these processes require hydrophobicity. Thus, that may explain the reason for the higher hydrophobicity threshold for Gram-negative bacteria. Overall, these studies indicate the relative threshold of hydrophobicity for Gram-negative bacteria is higher than Gram-positive bacteria. A previous study on cyclic peptides agrees that Gram-negative bacteria need a higher threshold related to antimicrobial activity [49]. During that study, it was generally accepted the hydrophobicity and the antibacterial activity had a positive correlation and increasing hydrophobicity of the polar and the nonpolar faces increased the antimicrobial activity [45,50].

A nonlinear relationship between hydrophobicity and antimicrobial activity was also mentioned in a V13K_L_ study, which had similar results [51]. The study attributed this phenomenon to the dimerisation of peptides. Although there are few reports on the hydrophobicity threshold, there are plenty of studies on the effect of dimerisation on the antibacterial ability of peptides. Previous research on the peptide Ctx-Ha and AU 1.2 found dimerisation decreases the antimicrobial activity [52,53]. Higher self-association would reduce dissociation ability and weaken the ability to penetrate cell membranes and cell walls, resulting in a decrease of antibacterial ability [51]. Meanwhile, the self-association hinders the peptide to cross the membrane or take more cost, resulting in the inhibition of its antibacterial activity [54].The self-association ability is related to the hydrophobicity [51,55,56], and that may explain the results for the B1A analogues in relation to the strength of hydrophobicity.

Taken together, these results reveal a delicate balance between net charge and hydrophobicity is necessary for broad-spectrum antimicrobial activity and reduced toxicity. An increase in hydrophobicity increases haemolysis. However, these two factors (net charge and hydrophobicity) no longer promote antimicrobial activity after reaching a certain level. The charges must be up to four, which is the charge of the parent peptide B1A. Meanwhile, the hydrophobicity should reach 0.806 for the best broad-spectrum antimicrobial activity, at least in this study. However, this balance becomes more difficult to maintain if you increase the variation from the parent peptide. 

## 5. Conclusions

In conclusion, structure–activity relationship studies of the Brevinin-1 peptide B1A were carried out via the rational design of analogues. The results of the bio-functional studies indicate the analogue KKWB2 appears to be the best candidate peptide due to its therapeutic index. Different thresholds for hydrophobicity relating to the antimicrobial activity against different bacteria were also identified to provide new insight into peptide design and structure–activity relationship studies.

## Figures and Tables

**Table 1:** The physicochemical properties of Brevinin-1 peptides and B1A’s analogues.

Peptide	Sequences	Residue No	Charge	Hydrophobicity <H>	Hydrophobic moment <µH>
B1PLB	FLPLIAGLAANFLPKIFCAITKKC	24	3	0.834	0.310
B1PLC	FLPVIAGVAAKFLPKIFCAITKKC	24	4	0.778	0.352
B1A	FLPLIAGLAAKFLPKIFCAITKKC	24	4	0.818	0.326
B1A1	FLPLIAGLAAKCAITKKC	18	3	0.712	0.246
B1A2	FLPKIFCAITKKC	13	3	0.791	0.552
KB2	KFLPKIFCAITKKC	14	4	0.644	0.577
KKB2	KKFLPKIFCAITKKC	15	5	0.553	0.505
KWB2	KFLPWKIFCAITKKC	15	4	0.789	0.285
KKWB2	KKFLPWKIFCAITKKC	16	5	0.659	0.255
KW^3,5^B2	KFWPWKIFCAITKKC	15	4	0.806	0.263
KW^5,7^B2	KFLPWKWFCAITKKC	15	4	0.799	0.287

**Table 2:** Secondary structure analysis of B1A and its analogues.

Peptide		H_2_O			1% SDS		
No	Name	Helix	Antiparallel	Turn	Helix	Antiparallel	Turn
1	B1A	10.1	40.3	4.1	66.7	1.4	3.8
2	B1A1	6.1	25.7	20.9	29.3	4.9	21.4
3	B1A2	5.1	30.7	16.1	27.5	1.6	15.3
4	KB2	10.1	21.3	17.3	20.8	9	15.2
5	KKB2	11.4	19.0	13.8	18	30.9	15.2
6	KWB2	11.7	23.0	14.9	20.7	13.1	15.2
7	KKWB2	10.6	28.1	15.4	18.5	13.4	14.0
8	KW^3,5^B2	6.3	33.7	14.0	10.3	38.3	11.2
9	KW^5,7^B2	4.8	31.9	14.3	20.9	9.9	16.7

**Table 3:** Minimum inhibitory concentration (MICs), minimum bactericidal concentration (MBCs), HC_10,_ and therapeutic indices (TIs) of B1A and its analogues.

	*S. aureus*	*E. coli*	*C. albicans*	MRSA	*E. faecalis*	*P. aeruginosa*	*K. pneumoniae*	HC_10_	TI
**B1A**	4/8	8/32	4/8	16/32	8/32	32/64	16/16	1.576	0.162
**B1A1**	512/>512	512/>512	512/512	>512/>512	>512/>512	>512/>512	>512/>512	>512	1
**B1A2**	256/256	256/256	512/512	256/>512	>512/>512	>512/>512	>512/>512	>512	1.682
**KB2**	8/64	32/64	128/512	16/128	512/>512	512/>512	256/256	154	1.788
**KKB2**	8/64	16/32	32/64	16/64	512/512	256/>512	128/256	207	3.943
**KWB2**	8/32	16/32	16/64	8/64	256/512	128/256	64/128	99.81	3.119
**KKWB2**	8/16	16/32	8/32	8/32	256/512	64/64	32/32	140.04	5.890
**KW^3,5^B2**	8/16	16/32	8/32	8/32	256/256	64/128	32/64	32.07	1.349
**KW^5,7^B2**	8/16	16/64	16/32	8/32	256/512	64/128	32/64	58.42	2.225

MICs/MBCs (µM). HC_10_ (µM). MRSA: methicillin-resistant *S. aureus*.

## Supplementary Materials

The following are available online at https://www.mdpi.com/2079-7737/9/8/209/s1, Figure S1: The RP-HPLC chromatograms and the mass spectra of all the synthetic peptides. B1A(a); B1A1(b); B1A2(c); KB2(d); KKB2(e); KWB2(f); KKWB2(g); KW3,5B2(h); KW5,7B2(i). The gradient of acetonitrile is indicated by the blueline.

## References

[1] Li B., Webster T.J. (2018). Bacteria antibiotic resistance: New challenges and opportunities for implant-associated orthopedic infections. J. Orthop. Res.^®^.

[2] Wang G., Li X., Wang Z. (2016). APD3: The antimicrobial peptide database as a tool for research and education. Nucleic Acids Res..

[3] Mahlapuu M., Håkansson J., Ringstad L., Björn C. (2016). Antimicrobial peptides: An emerging category of therapeutic agents. Front. Cell. Infect. Microbiol..

[4] Reddy K., Yedery R., Aranha C. (2004). Antimicrobial peptides: Premises and promises. Int. J. Antimicrob. Agents.

[5] Xi X., Li B., Chen T., Kwok H.F. (2015). A review on bradykinin-related peptides isolated from amphibian skin secretion. Toxins.

[6] Lee J.-K., Luchian T., Park Y. (2018). New antimicrobial peptide kills drug-resistant pathogens without detectable resistance. Oncotarget.

[7] Savelyeva A., Ghavami S., Davoodpour P., Asoodeh A., Łos M.J. (2014). An Overview of Brevinin Superfamily: Structure, Function and Clinical Perspectives. Anticancer Genes.

[8] Novković M., Simunić J., Bojović V., Tossi A., Juretić D. (2012). DADP: The database of anuran defense peptides. Bioinformatics.

[9] Clark D.P., Durell S., Maloy W.L., Zasloff M. (1994). Ranalexin. A novel antimicrobial peptide from bullfrog (Rana catesbeiana) skin, structurally related to the bacterial antibiotic, polymyxin. J. Biol. Chem..

[10] Kwon M.-Y., Hong S.-Y., Lee K.-H. (1998). Structure-activity analysis of brevinin 1E amide, an antimicrobial peptide from Rana esculenta. Biochim. et Biophys. Acta (BBA) Protein Struct. Mol. Enzymol..

[11] Abraham P., Anand Sundaram A.R., Reshmy V., George S., Kumar K.S. (2015). Structure-activity relationship and mode of action of a frog secreted antibacterial peptide B1CTcu5 using synthetically and modularly modified or deleted (SMMD) peptides. PLoS ONE.

[12] Barra D., Simmaco M. (1995). Amphibian skin: A promising resource for antimicrobial peptides. Trends Biotechnol..

[13] Kumari V., Nagaraj R. (2001). Structure—Function studies on the amphibian peptide brevinin 1E: Translocating the cationic segment from the C-terminal end to a central position favors selective antibacterial activity. J. Pept. Res..

[14] Conlon J.M., Kolodziejek J., Nowotny N. (2004). Antimicrobial peptides from ranid frogs: Taxonomic and phylogenetic markers and a potential source of new therapeutic agents. Biochim. et Biophys. Acta.

[15] Basir Y.J., Knoop F.C., Dulka J., Conlon J.M. (2000). Multiple antimicrobial peptides and peptides related to bradykinin and neuromedin N isolated from skin secretions of the pickerel frog, Rana palustris. Biochim. et Biophys. Acta (BBA) Protein Struct. Mol. Enzymol..

[16] Zhou M., Wang L., Owens D.E., Chen T., Walker B., Shaw C. (2007). Rapid identification of precursor cDNAs encoding five structural classes of antimicrobial peptides from pickerel frog (Rana palustris) skin secretion by single step “shotgun” cloning. Peptides.

[17] Lin Y., Hu N., He H., Ma C., Zhou M., Wang L., Chen T. (2019). A hylarana latouchii skin secretion-derived novel bombesin-related pentadecapeptide (ranatensin-HLa) evoke myotropic effects on the in vitro rat smooth muscles. Toxins.

[18] Sidorova M., Molokoedov A., Az′muko A., Kudryavtseva E., Krause E., Ovchinnikov M., Bespalova Z.D. (2004). The use of hydrogen peroxide for closing disulfide bridges in peptides. Russ. J. Bioorganic Chem..

[19] Gautier R., Douguet D., Antonny B., Drin G. (2008). HELIQUEST: A web server to screen sequences with specific α-helical properties. Bioinformatics.

[20] Micsonai A., Wien F., Kernya L., Lee Y.H., Goto Y., Réfrégiers M., Kardos J. (2015). Accurate secondary structure prediction and fold recognition for circular dichroism spectroscopy. Proc. Natl. Acad. Sci. USA.

[21] Yang J., Zhang Y. (2015). I-TASSER server: New development for protein structure and function predictions. Nucleic Acids Res..

[22] Gao Y., Wu D., Xi X., Wu Y., Ma C., Zhou M., Wang L., Yang M., Chen T., Shaw C. (2016). Identification and characterisation of the antimicrobial peptide, phylloseptin-PT, from the skin secretion of Phyllomedusa tarsius, and comparison of activity with designed, cationicity-enhanced analogues and diastereomers. Molecules.

[23] Gao Y., Wu D., Wang L., Lin C., Ma C., Xi X., Zhou M., Duan J., Bininda-Emonds O.R., Chen T. (2017). Targeted modification of a novel amphibian antimicrobial peptide from Phyllomedusa tarsius to enhance its activity against MRSA and microbial biofilm. Front. Microbiol..

[24] Kolari M., Rautiainen J., Hentze H.-P., Alakomi H.-L., Forssell P. (2015). Biocide Formulation and Method for Treating Water. U.S. Patent.

[25] Loh B., Grant C., Hancock R. (1984). Use of the fluorescent probe 1-N-phenylnaphthylamine to study the interactions of aminoglycoside antibiotics with the outer membrane of pseudomonas aeruginosa. Antimicrob. Agents Chemother..

[26] Skerlavaj B., Romeo D., Gennaro R. (1990). Rapid membrane permeabilization and inhibition of vital functions of gram-negative bacteria by bactenecins. Infect. Immun..

[27] Lehrer R., Barton A., Daher K.A., Harwig S., Ganz T., Selsted M.E. (1989). Interaction of human defensins with *Escherichia coli*. Mechanism of bactericidal activity. J. Clin. Investig..

[28] Zohrab F., Askarian S., Jalili A., Oskuee R.K. (2019). Biological properties, current applications and potential therapeautic applications of brevinin peptide superfamily. Int. J. Pept. Res. Ther..

[29] Yeaman M.R., Yount N.Y. (2003). Mechanisms of antimicrobial peptide action and resistance. Pharmacol. Rev..

[30] Won H.-S., Kang S.-J., Lee B.-J. (2009). Action mechanism and structural requirements of the antimicrobial peptides, gaegurins. Biochim. et Biophys. Acta (BBA) Biomembr..

[31] Suh J.-Y., Lee K.-H., Chi S.-W., Hong S.-Y., Choi B.-W., Moon H.-M., Choi B.-S. (1996). Unusually stable helical kink in the antimicrobial peptide—A derivative of gaegurin. FEBS Lett..

[32] Wang J., Dou X., Song J., Lyu Y., Zhu X., Xu L., Li W., Shan A. (2019). Antimicrobial peptides: Promising alternatives in the post feeding antibiotic era. Med. Res. Rev..

[33] Åmand H.L., Fant K., Nordén B., Esbjörner E.K. (2008). Stimulated endocytosis in penetratin uptake: Effect of arginine and lysine. Biochem. Biophys. Res. Commun..

[34] Situ A.J., Kang S.-M., Frey B.B., An W., Kim C., Ulmer T.S. (2018). Membrane anchoring of α-helical proteins: Role of tryptophan. J. Phys. Chem. B.

[35] Arias M., Nguyen L.T., Kuczynski A.M., Lejon T., Vogel H.J. (2014). Position-dependent influence of the three trp residues on the membrane activity of the antimicrobial peptide, tritrpticin. Antibiotics.

[36] Rekdal Ø., Haug B.E., Kalaaji M., Hunter H.N., Lindin I., Israelsson I., Solstad T., Yang N., Brandl M., Mantzilas D. (2012). Relative spatial positions of tryptophan and cationic residues in helical membrane-active peptides determine their cytotoxicity. J. Biol. Chem..

[37] de Planque M.R., Goormaghtigh E., Greathouse D.V., Koeppe R.E., Kruijtzer J.A., Liskamp R.M., de Kruijff B., Killian J.A. (2001). Sensitivity of single membrane-spanning α-helical peptides to hydrophobic mismatch with a lipid bilayer: Effects on backbone structure, orientation, and extent of membrane incorporation. Biochemistry.

[38] Schibli D.J., Epand R.F., Vogel H.J., Epand R.M. (2002). Tryptophan-rich antimicrobial peptides: Comparative properties and membrane interactions. Biochem. Cell Biol..

[39] Bi X., Wang C., Ma L., Sun Y., Shang D. (2013). Investigation of the role of tryptophan residues in cationic antimicrobial peptides to determine the mechanism of antimicrobial action. J. Appl. Microbiol..

[40] Mishra A.K., Choi J., Moon E., Baek K.-H. (2018). Tryptophan-rich and proline-rich antimicrobial peptides. Molecules.

[41] Le C.-F., Fang C.-M., Sekaran S.D. (2017). Intracellular targeting mechanisms by antimicrobial peptides. Antimicrob. Agents Chemother..

[42] Ehrenstein G., Lecar H. (1977). Electrically gated ionic channels in lipid bilayers. Q. Rev. Biophys..

[43] Shai Y. (1999). Mechanism of the binding, insertion and destabilization of phospholipid bilayer membranes by α-helical antimicrobial and cell non-selective membrane-lytic peptides. Biochim. et Biophys. Acta (BBA) Biomembr..

[44] Tachi T., Epand R.F., Epand R.M., Matsuzaki K. (2002). Position-dependent hydrophobicity of the antimicrobial magainin peptide affects the mode of peptide—Lipid interactions and selective toxicity. Biochemistry.

[45] Avrahami D., Shai Y. (2002). Conjugation of a magainin analogue with lipophilic acids controls hydrophobicity, solution assembly, and cell selectivity. Biochemistry.

[46] Blondelle S.E., Houghten R.A. (1991). Hemolytic and antimicrobial activities of the twenty-four individual omission analogs of melittin. Biochemistry.

[47] Silhavy T.J., Kahne D., Walker S. (2010). The bacterial cell envelope. Cold Spring Harb. Perspect. Biol..

[48] Dean R., Van Kan J.A., Pretorius Z.A., Hammond-Kosack K.E., Di Pietro A., Spanu P.D., Rudd J.J., Dickman M., Kahmann R., Ellis J. (2012). The Top 10 fungal pathogens in molecular plant pathology. Mol. Plant Pathol..

[49] Kondejewski L.H., Lee D.L., Jelokhani-Niaraki M., Farmer S.W., Hancock R.E., Hodges R.S. (2002). Optimization of microbial specificity in cyclic peptides by modulation of hydrophobicity within a defined structural framework. J. Biol. Chem..

[50] Dathe M., Wieprecht T., Nikolenko H., Handel L., Maloy W.L., MacDonald D.L., Beyermann M., Bienert M. (1997). Hydrophobicity, hydrophobic moment and angle subtended by charged residues modulate antibacterial and haemolytic activity of amphipathic helical peptides. FEBS Lett..

[51] Chen Y., Guarnieri M.T., Vasil A.I., Vasil M.L., Mant C.T., Hodges R.S. (2007). Role of peptide hydrophobicity in the mechanism of action of α-helical antimicrobial peptides. Antimicrob. Agents Chemother..

[52] Lorenzón E., Cespedes G., Vicente E., Nogueira L., Bauab T., Castro M., Cilli E.M. (2012). Effects of dimerization on the structure and biological activity of antimicrobial peptide Ctx-Ha. Antimicrob. Agents Chemother..

[53] Lorenzon E., Sanches P., Nogueira L., Bauab T., Cilli E.M. (2013). Dimerization of aurein 1.2: Effects in structure, antimicrobial activity and aggregation of Candida albicans cells. Amino Acids.

[54] Zou R., Zhu X., Tu Y., Wu J., Landry M.P. (2018). Activity of antimicrobial peptide aggregates decreases with increased cell membrane embedding free energy cost. Biochemistry.

[55] Mant C.T., Chen Y., Hodges R.S. (2003). Temperature profiling of polypeptides in reversed-phase liquid chromatography: I. Monitoring of dimerization and unfolding of amphipathic α-helical peptides. J. Chromatogr. A.

[56] Mant C.T., Tripet B., Hodges R.S. (2003). Temperature profiling of polypeptides in reversed-phase liquid chromatography: II. Monitoring of folding and stability of two-stranded α-helical coiled-coils. J. Chromatogr. A.

