# Supplementary material for: Modification and Targeted Design of N-Terminal Truncates Derived from Brevinin with Improved Therapeutic Efficacy
Source: Biology (Basel). 2020 Aug 6;9(8):209. doi: 10.3390/biology9080209 (PMC7464788; doi:10.3390/biology9080209)
Supplement: Supplementary file 1 [file biology-09-00209-s001.pdf]

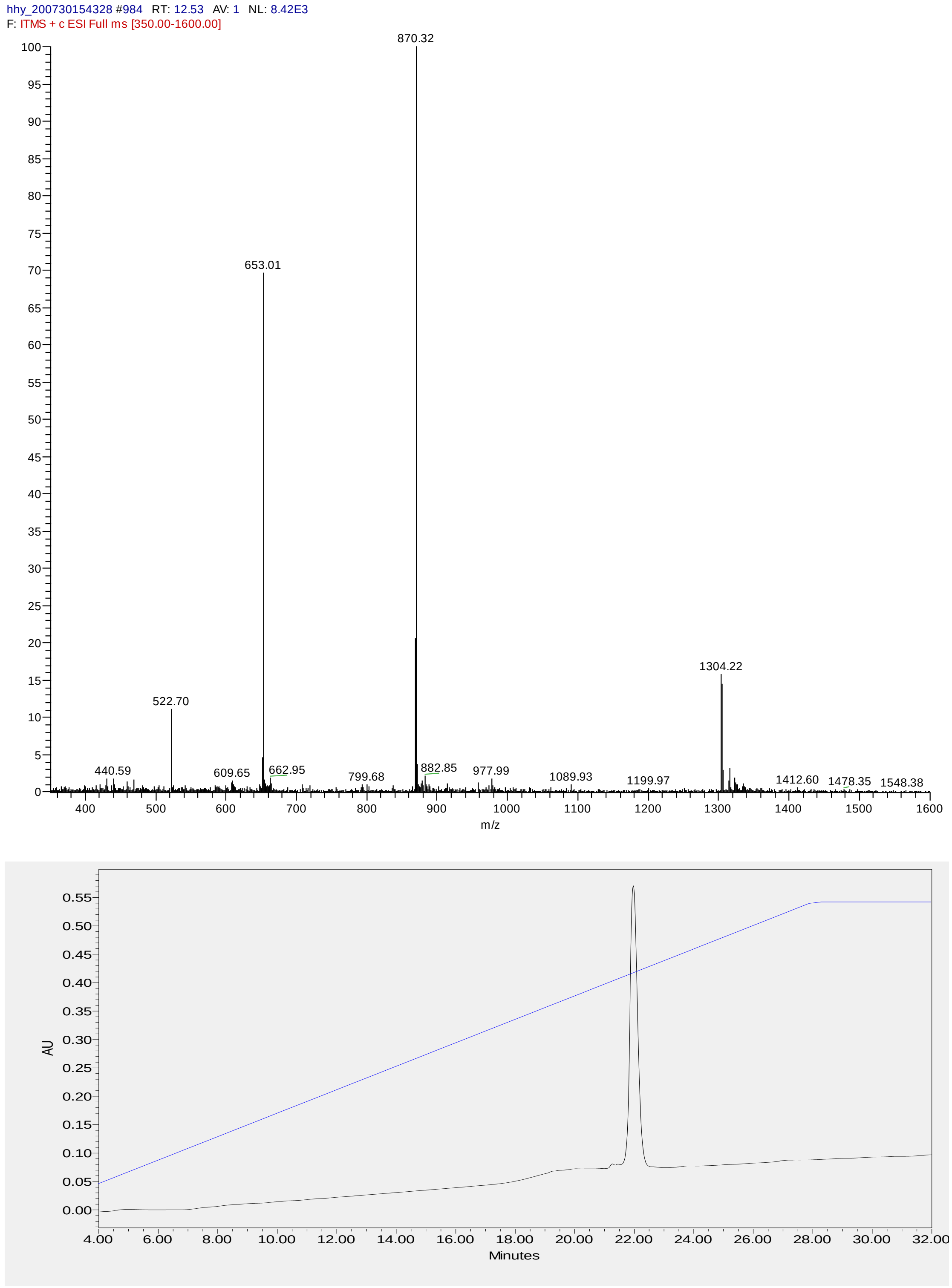
hhy_200730154328 #984 RT: 12.53 AV: 1 NL: 8.42E3
F: ITMS + c ESI Full ms [350.00-1600.00]
870.32
653.01
522.70
1304.22
440.59
609.65
662.95
799.68
882.85
977.99
1089.93
1199.97
1412.60
1478.35
1548.38
m/z
AU
Minutes

(**a**)

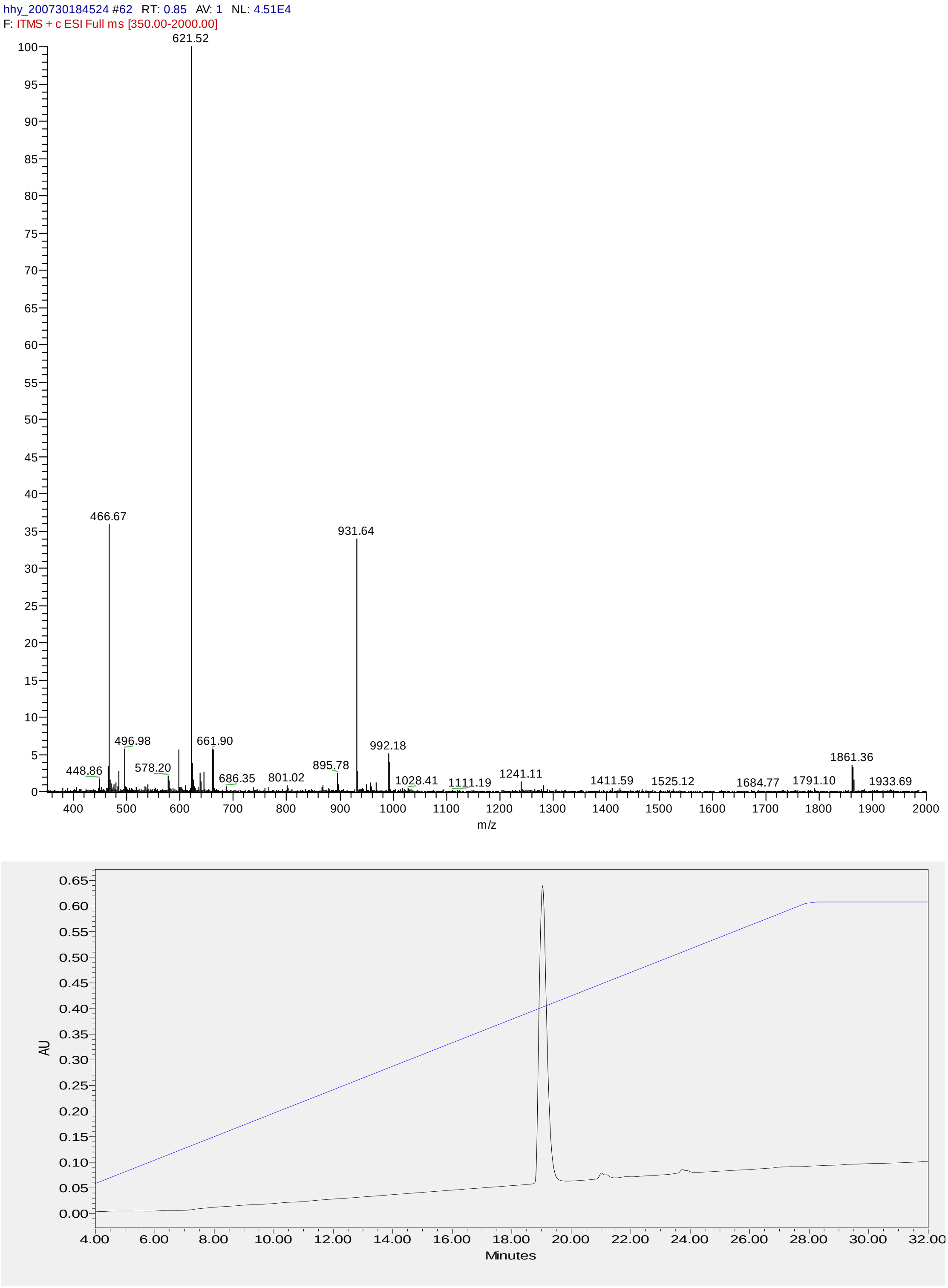
hhy_200730184524 #62 RT: 0.85 AV: 1 NL: 4.51E4
F: ITMS + c ESI Full ms [350.00-2000.00]
621.52
466.67
931.64
496.98
661.90
992.18
1861.36
448.86
578.20
686.35
801.02
895.78
1028.41
1111.19
1241.11
1411.59
1525.12
1684.77
1791.10
1933.69
m/z
AU
Minutes

(**b**)

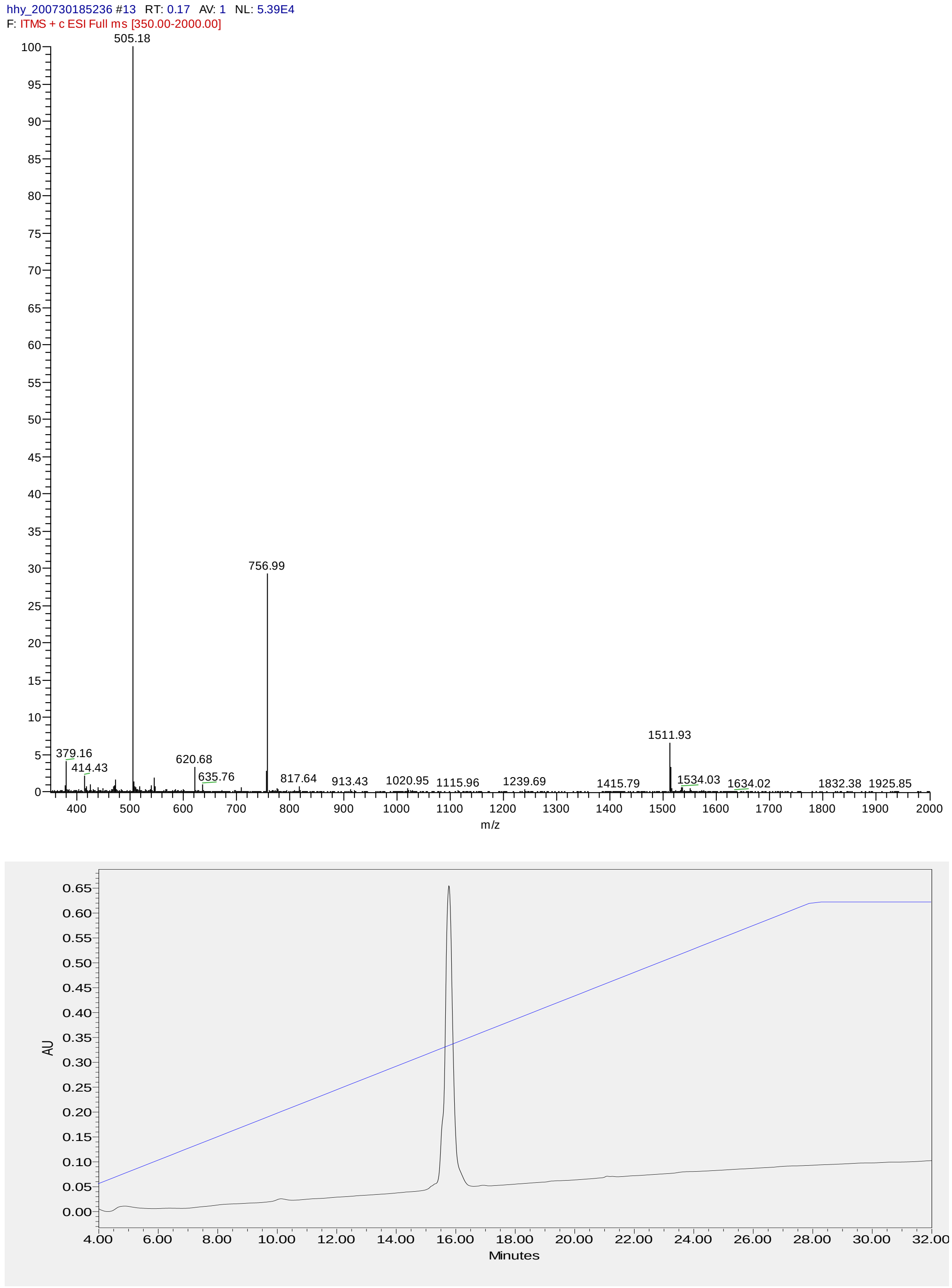
hhy_200730185236 #13 RT: 0.17 AV: 1 NL: 5.39E4
F: ITMS + c ESI Full ms [350.00-2000.00]
505.18
756.99
1511.93
379.16
414.43
620.68
635.76
817.64
913.43
1020.95
1115.96
1239.69
1415.79
1534.03
1634.02
1832.38
1925.85
m/z
AU
Minutes

(c)

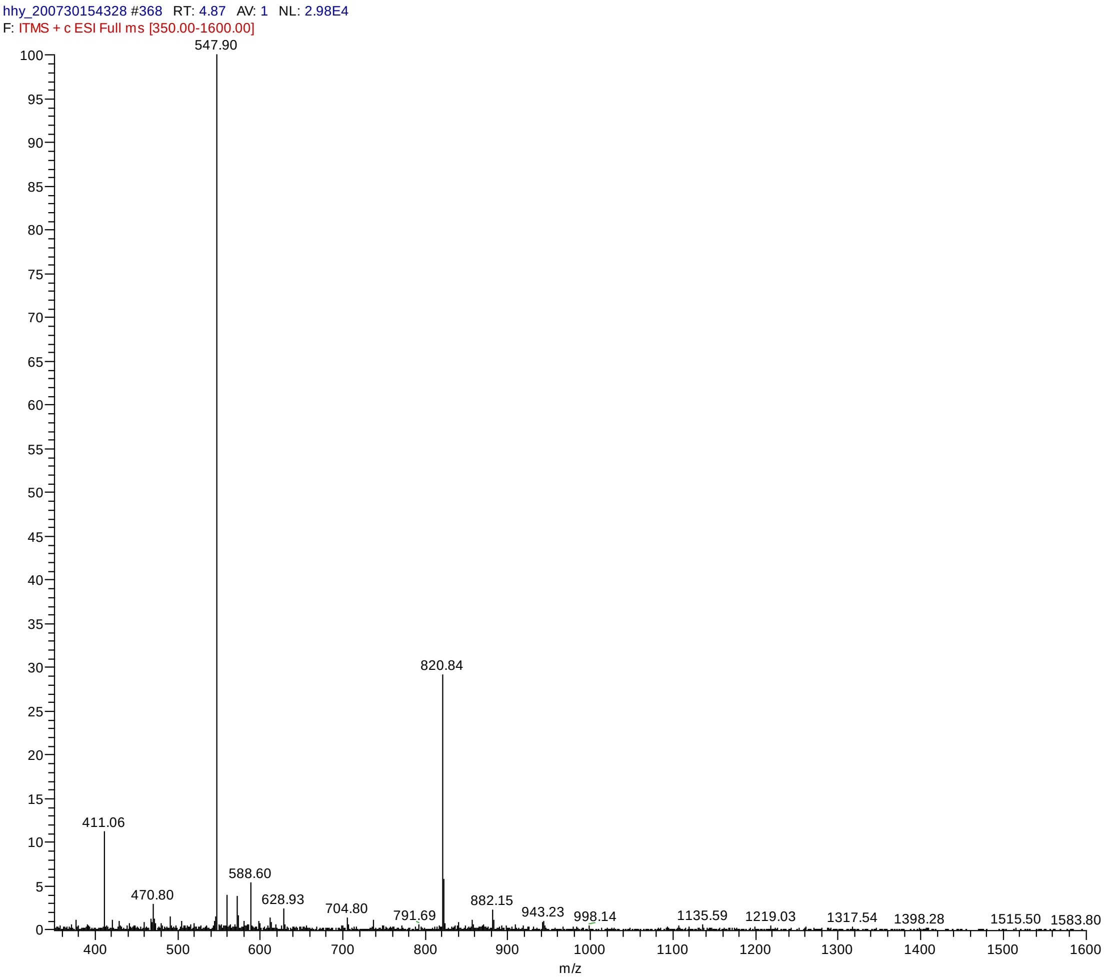
hhy_200730154328 #368 RT: 4.87 AV: 1 NL: 2.98E4
F: ITMS + c ESI Full ms [350.00-1600.00]
547.90
820.84
411.06
470.80
588.60
628.93
704.80
791.69
882.15
943.23
998.14
1135.59
1219.03
1317.54
1398.28
1515.50
1583.80
m/z

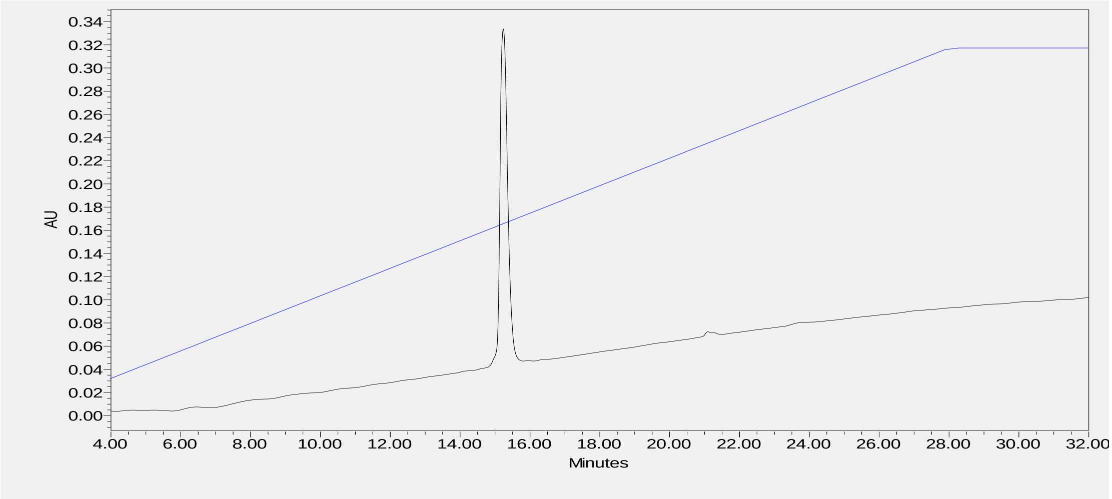
AU
Minutes

(**d**)

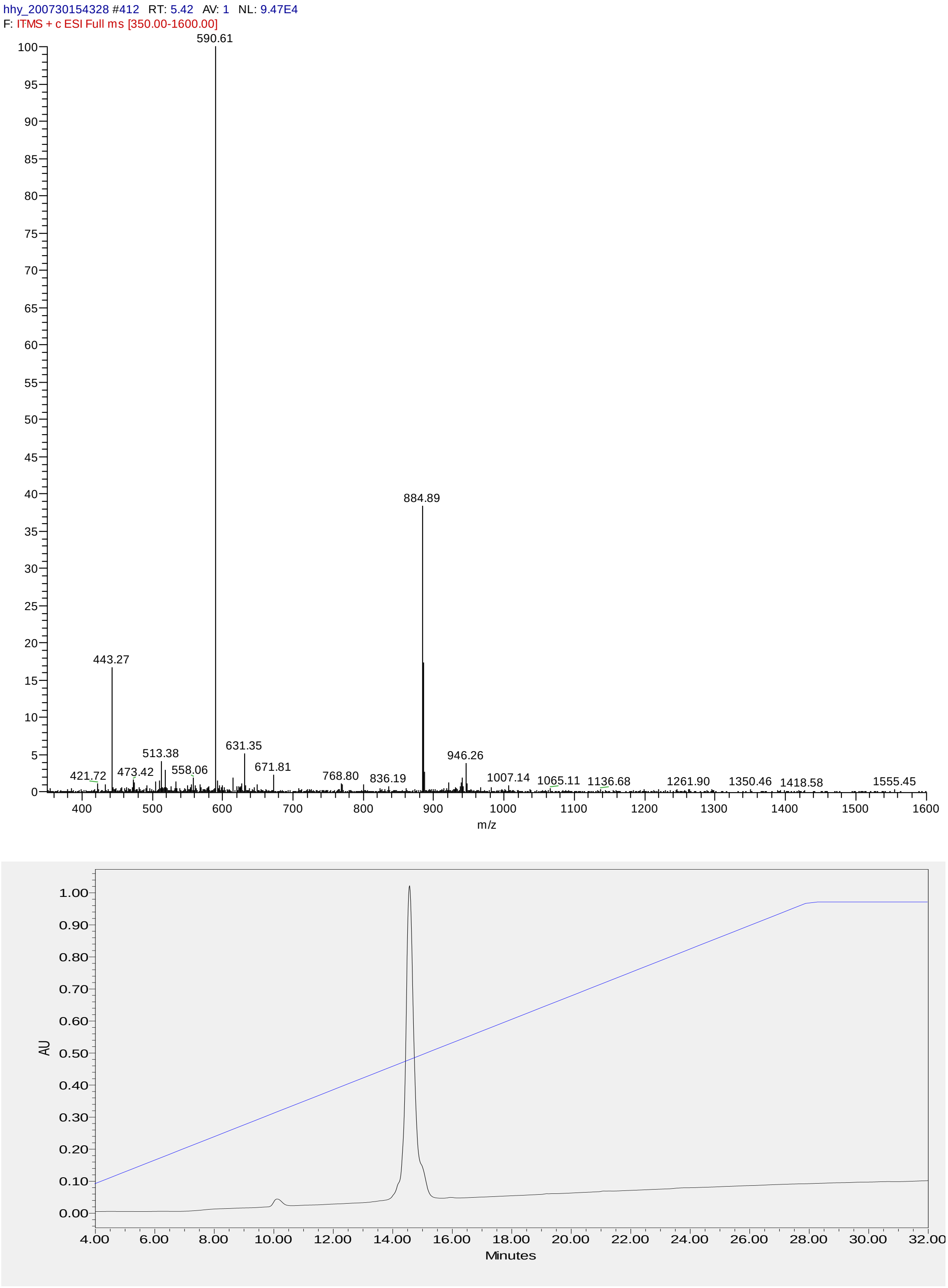
hhy_200730154328 #412 RT: 5.42 AV: 1 NL: 9.47E4
F: ITMS + c ESI Full ms [350.00-1600.00]
590.61
884.89
443.27
513.38
631.35
671.81
946.26
421.72
473.42
558.06
768.80
836.19
1007.14
1065.11
1136.68
1261.90
1350.46
1418.58
1555.45
m/z
AU
Minutes

(e)

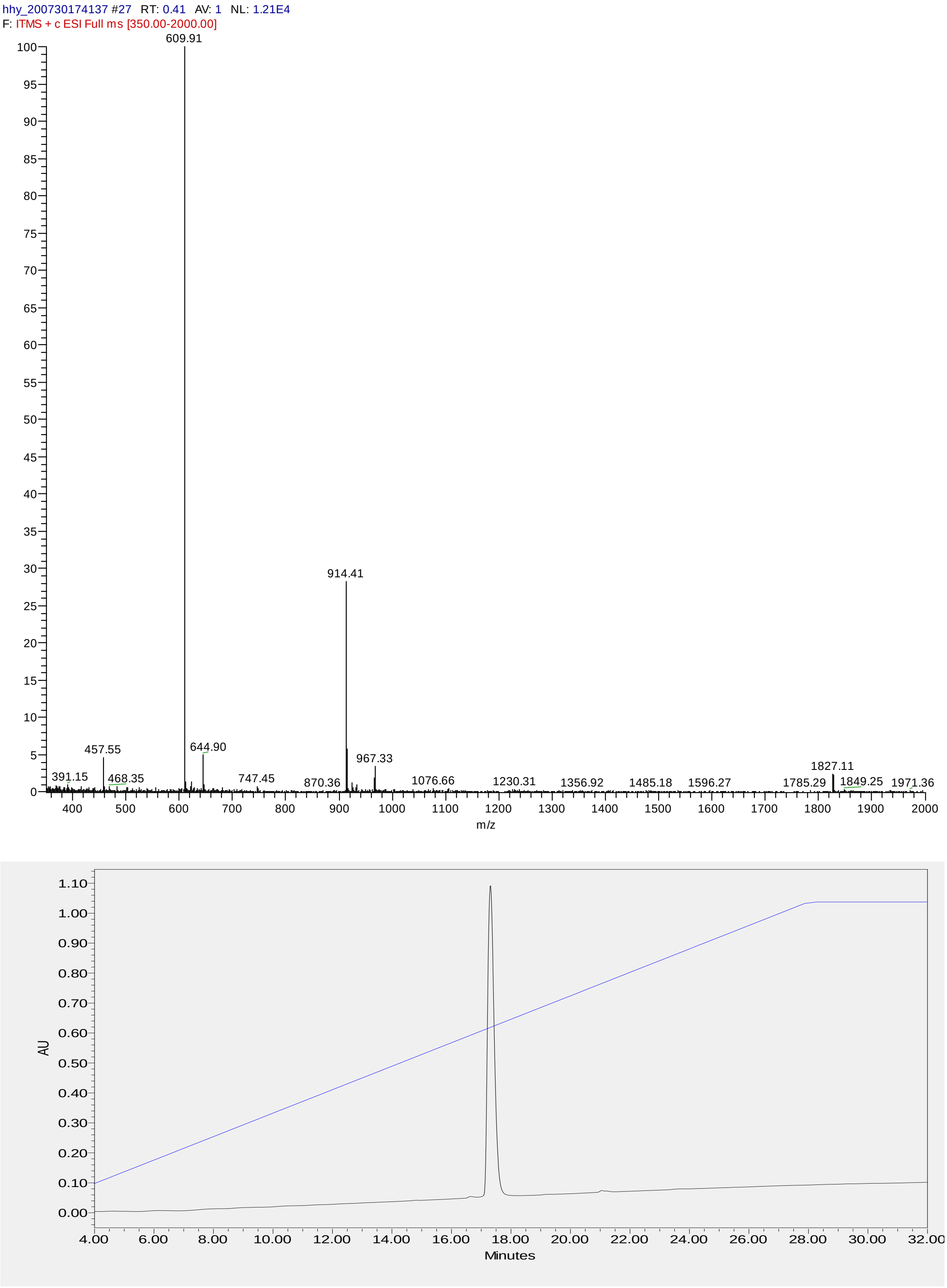
hhy_200730174137 #27 RT: 0.41 AV: 1 NL: 1.21E4
F: ITMS + c ESI Full ms [350.00-2000.00]
609.91
914.41
457.55
644.90
967.33
391.15
468.35
747.45
870.36
1076.66
1230.31
1356.92
1485.18
1596.27
1785.29
1827.11
1849.25
1971.36
m/z
AU
Minutes

(f)

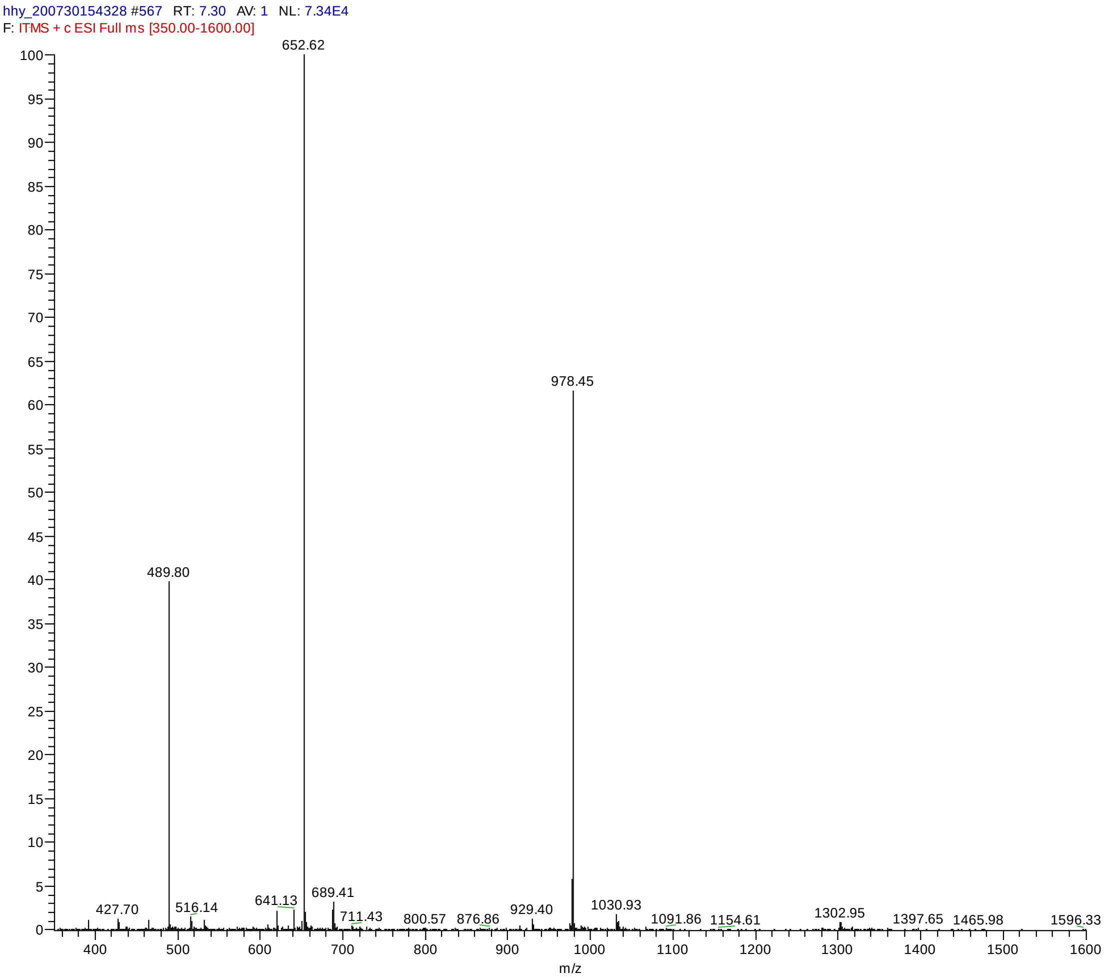
hhy_200730154328 #567 RT: 7.30 AV: 1 NL: 7.34E4
F: ITMS + c ESI Full ms [350.00-1600.00]
652.62
978.45
489.80
427.70
516.14
641.13
689.41
711.43
800.57
876.86
929.40
1030.93
1091.86
1154.61
1302.95
1397.65
1465.98
1596.33
m/z

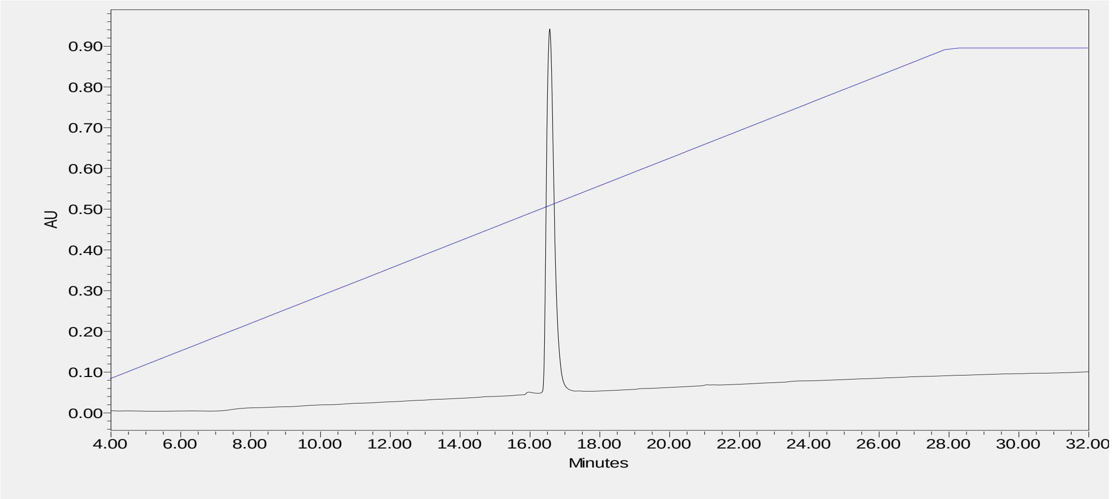
AU
Minutes

(g)

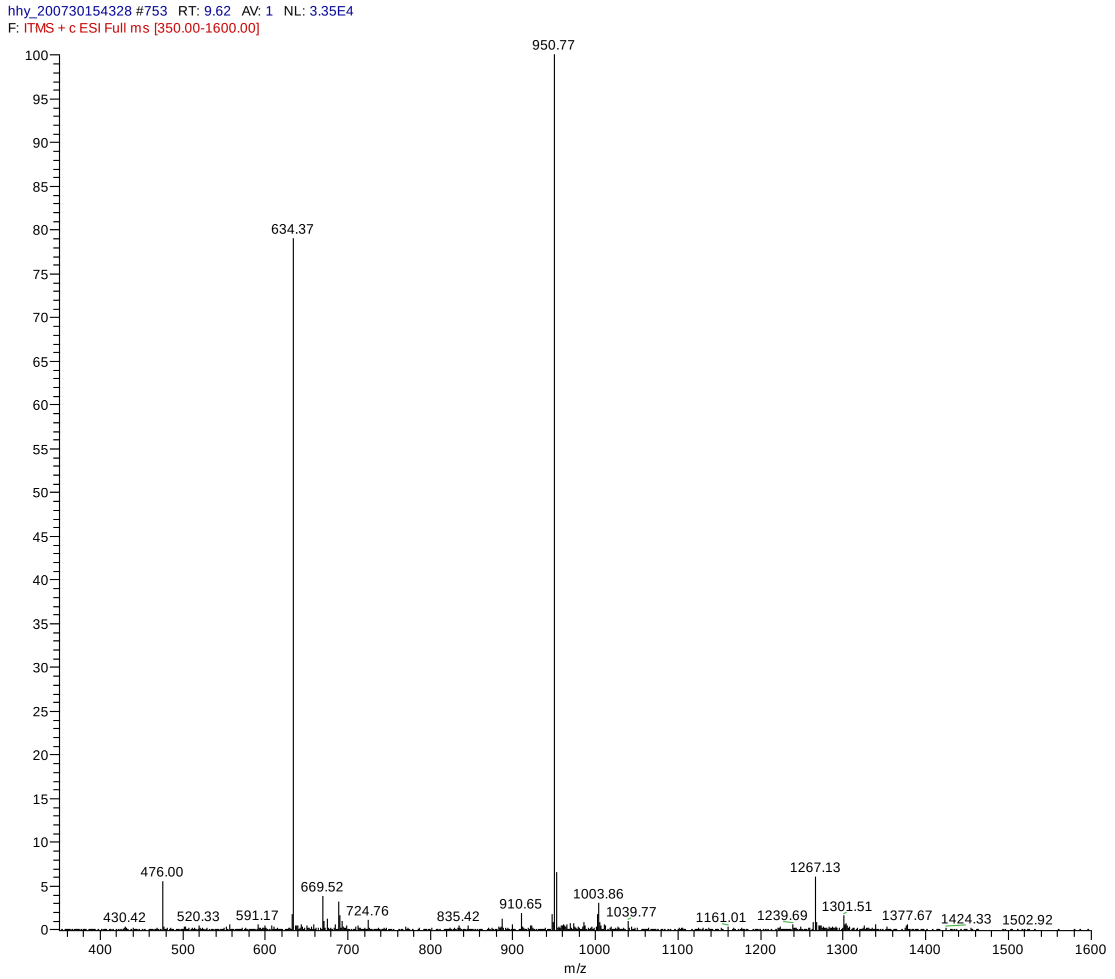
hhy_200730154328 #753 RT: 9.62 AV: 1 NL: 3.35E4
F: ITMS + c ESI Full ms [350.00-1600.00]
950.77
634.37
476.00
669.52
724.76
430.42
520.33
591.17
835.42
910.65
1003.86
1039.77
1161.01
1239.69
1267.13
1301.51
1377.67
1424.33
1502.92
m/z

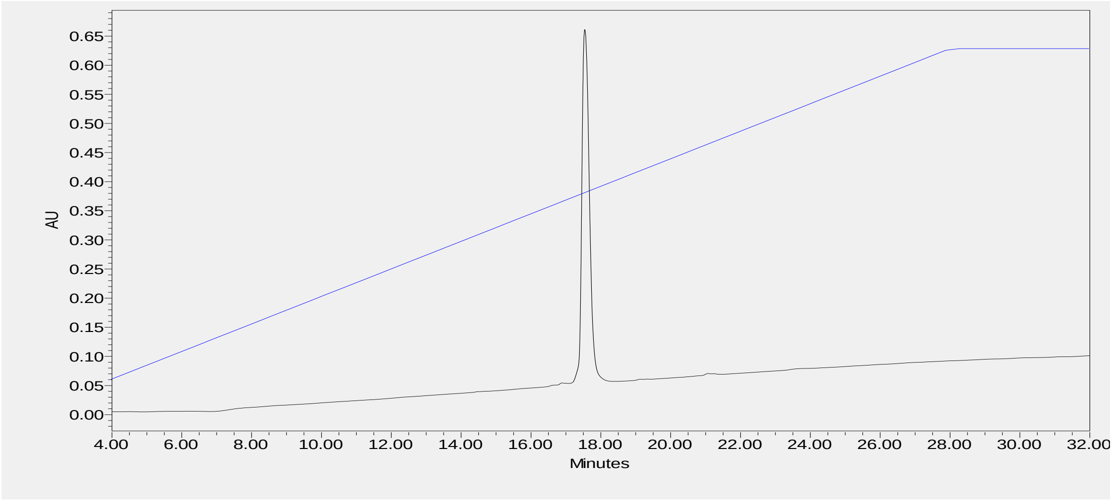
AU
Minutes

(h)

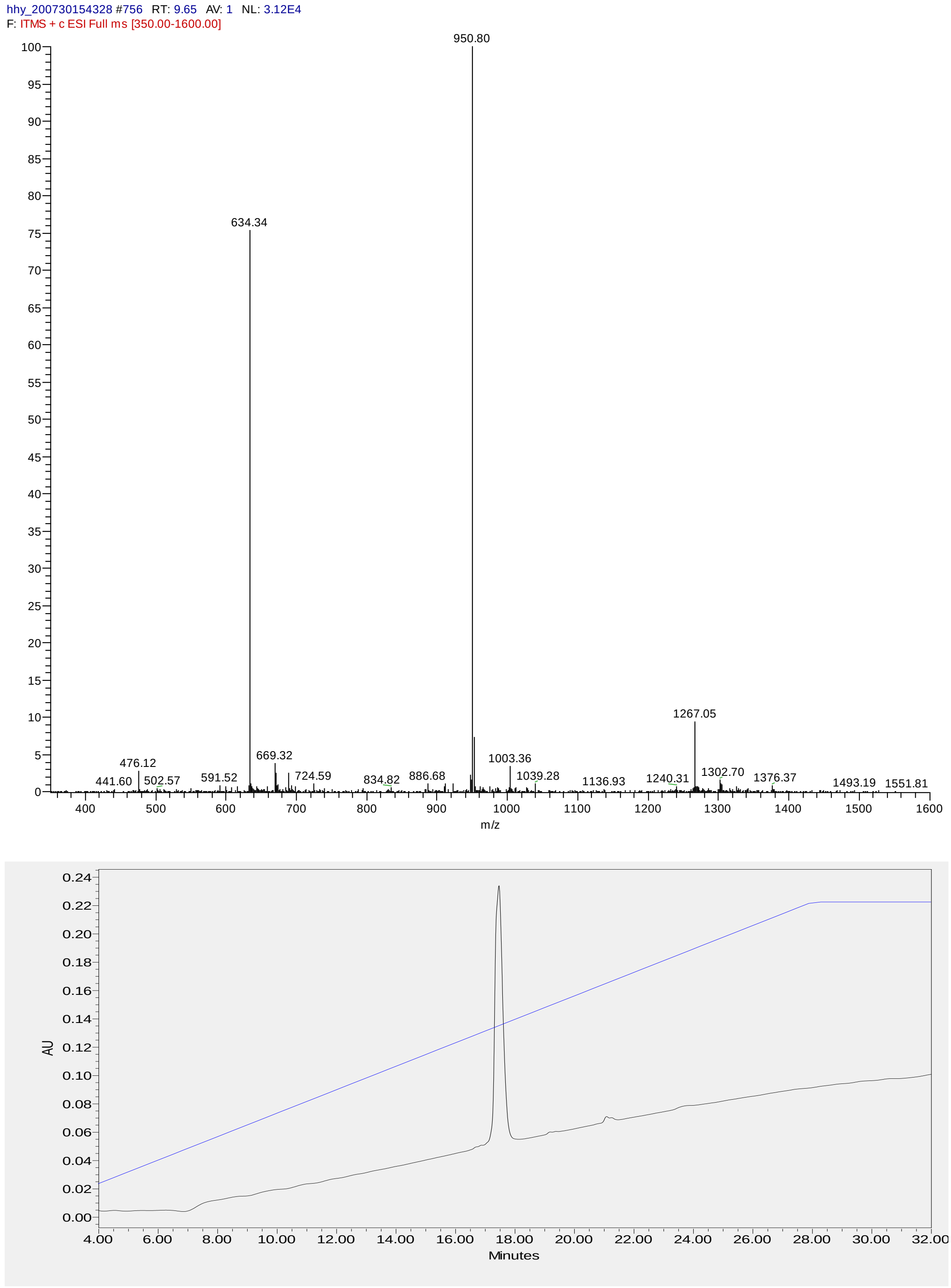

(i)

Figure S1. The RP-HPLC chromatograms and the mass spectra of all the synthetic peptides: B1A(**a**); B1A1(**b**); B1A2(**c**); KB2(**d**); KKB2(**e**); KWB2(**f**); KKWB2(**g**); KW^{3,5}B2(**h**); KW^{5,7}B2(**i**). The gradient of acetonitrile is indicated by the blueline.
